# Anisotropic Ti_*x*_Sn_1-__*x*_O_2 _nanostructures prepared by magnetron sputter deposition

**DOI:** 10.1186/1556-276X-6-326

**Published:** 2011-04-13

**Authors:** Shutian Chen, Zhengcao Li, Zhengjun Zhang

**Affiliations:** 1 State Key Laboratory of New Ceramic and Fine Processing, Department of Materials Science and Engineering, Tsinghua University, Beijing 100084, China

## Abstract

Regular arrays of Ti_*x*_Sn_1-__*x*_O_2 _nanoflakes were fabricated through glancing angle sputter deposition onto self-assembled close-packed arrays of 200-nm-diameter polystyrene spheres. The morphology of nanostructures could be controlled by simply adjusting the sputtering power of the Ti target. The reflectance measurements showed that the melon seed-shaped nanoflakes exhibited optimal properties of antireflection in the entire visible and ultraviolet region. In addition, we determined their anisotropic reflectance in the direction parallel to the surface of nanoflakes and perpendicular to it, arising from the anisotropic morphology.

## Introduction

Much research has recently been focused on the design of nanoscale semiconductor oxide materials with controlled morphology and novel morphology-dependent physical properties. As an n-type semiconductor oxide with a wide band gap (*E*_g _= 3.6 eV at 300 K), SnO_2 _is well known for its potential applications in gas sensing [1], photoconductors [2], photocatalysis [3], and dye-sensitized solar cells [4]. Similar to other highly transparent semiconductors, SnO_2 _is also expected to be a competitive candidate for optical devices [5]. To optimize the optical properties, tuning the composition and tailoring the morphology are very important.

Numerous techniques have been developed to synthesize SnO_2 _films. The advantage of magnetron sputter deposition lies in the ease and flexibility of doping, which is an effective method to adjust the composition, and to ensure good homogeneity and repeatability. Ti is an appealing alternative for doping, compared to other elements quantities of which are restricted due to the emergence of the second phase. Owing to the isostructure (rutile type) and slightly different lattice parameters of TiO_2 _and SnO_2_, the metal cations can replace each other in a wide concentration region [6].

On the other hand, the morphology of films can be modulated by the glancing angle deposition (GLAD) technique. The GLAD technique, which exploits the shadowing effects, is applied to fabricating a variety of nanostructures, such as columns, helixes, and springs [7-11]. With the combination of GLAD and magnetron sputter deposition techniques, arrays of uniquely shaped nanostructures built from a wide range of material systems can be created. However, the preparation of oxides by glancing angle sputter deposition is rarely reported, as the sputtering of oxide targets is hard to maintain in the pressure technically required for GLAD. In this study, periodic arrays of Ti_*x*_Sn_1-__*x*_O_2 _nanostructures were grown on patterned Si substrates using GLAD with the simultaneous deposition of DC and RF sputtering sources. The RF sputtering of the SnO_2 _target at low pressures resulted owing to the DC sputtering of the Ti target. The morphology of nanostructures can be modulated by the regulation of the sputtering power.

## Methods

**Figure 1:**
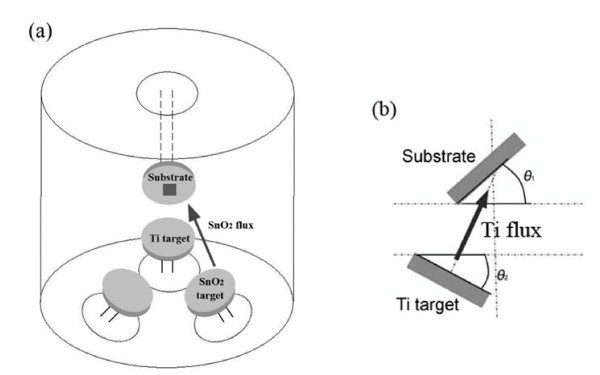
**Schematic illustration of the relative positions of the substrates, Ti targets and SnO_2 _target from (a) the bird's eye view and (b) the side view**.

The Ti_*x*_Sn_1-__*x*_O_2 _nanostructures were prepared on Si(001) substrates that were patterned using 200-nm-diameter hexagonal close-packed polystyrene microspheres in an ultrahigh vacuum magnetron sputter deposition system. Ti_*x*_Sn_1-__*x*_O_2 _depositions were carried out using a 6-cm-diameter Ti target (99.99% pure) and a 6-cm-diameter SnO_2 _target (99.9% pure) mounted at an angle of 120° with respect to each other, with the substrate backed against the Ti target, as shown in Figure 1a, b. The deposition angle *α*, equaling *θ*_1 _+ *θ*_2 _(defined in Figure 1b), was selected as 85°. Sputtering was carried out at 0.15 Pa which was held constant during all depositions in 99.999% pure Ar. No external substrate heating was applied. Power-regulated DC and RF power supplies were employed to provide the discharge currents of 0.15, 0.20, and 0.25 A, respectively (for three samples, 1#, 2#, and 3#) at 340 V for Ti, and a radio frequency current of 130 mA at 500 V for SnO_2_. The Ti_*x*_Sn_1-__*x*_O_2 _nanostructures were obtained with simultaneous deposition from sputtering sources like Ti and SnO_2 _onto a stationary substrate. The morphology of all the samples was examined by scanning electron microscopy (SEM), with their structure identified by X-ray diffraction (XRD) analysis, and the reflectance measured with a spectrophotometer.

## Results and discussion

**Figure 2:**
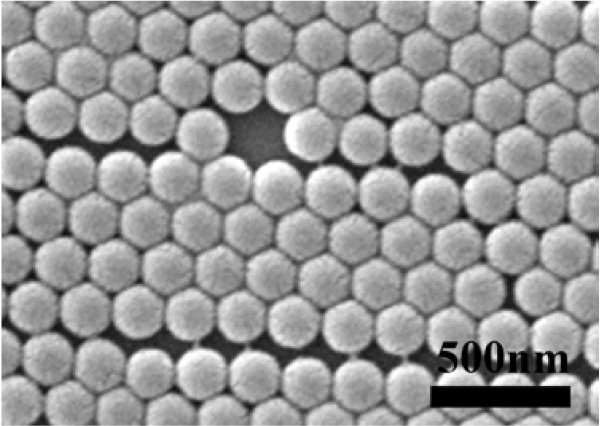
**Scanning electron micrograph from a close-packed array of 200-nm-diameter polystyrene spheres on Si (001) substrates**.

Figure 2 shows the typical SEM micrograph of the Si(001) substrate which is covered with a regular, hexagonal close-packed array of 200-nm-diameter polystyrene spheres. The polystyrene sphere layer exhibits various crystalline defects, including the vacancy, dislocation, and grain boundary, as illustrated in this figure. The formation of defects is attributed to a combination of the nanosphere's polydispersity and kinetic limitations during the drying process.

**Figure 3:**
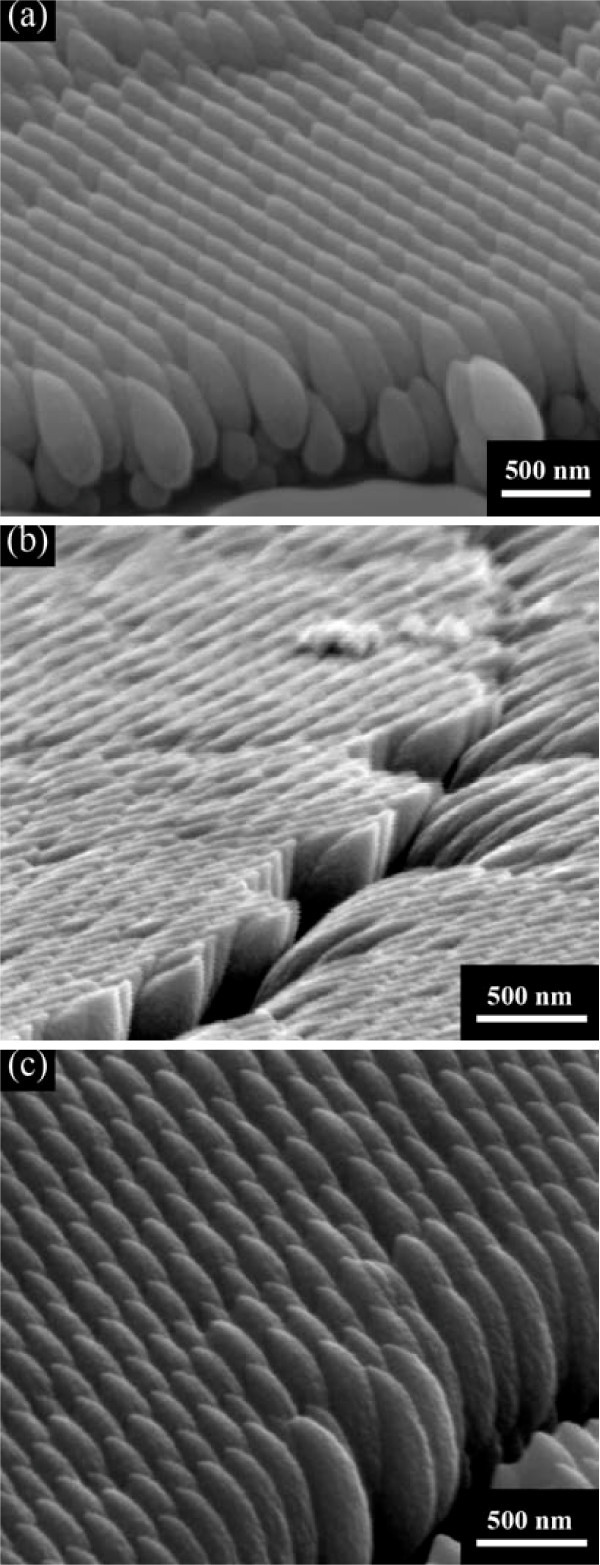
**Scanning electron microscopic images of Ti_*x*_Sn_1-__*x*_O_2 _films deposited at various discharge current (I) of Ti target**: (a) 0.15 A; (b) 0.20 A; and (c) 0.25 A.

For the deposition step, the power of RF sputtering was fixed, while the power of DC sputtering was regulated at the discharge currents 0.15, 0.20, and 0.25 A, respectively, for the three prepared samples named as 1#, 2#, and 3#. Their structures were identified by XRD, indicating an amorphous state in all the samples. As for the morphology, Figure 3 shows typical SEM micrographs of 1#, 2#, and 3#. Arrays of well-separated Ti_*x*_Sn_1-__*x*_O_2 _nanostructures were produced. The regular hexagonal arrays replicate the close-packed pattern of the polystyrene spheres, which indicates that each nanosphere leads to the formation of a nanostructure. It illustrates that substrate patterning is effective in positioning GLAD nanostructures into ordered arrays. Atomic shadowing and adatom diffusion are the dominant growth mechanisms in the process of GLAD. Oblique angle flux incidence enhances atomic shadowing which produces areas that vapor flux cannot directly reach while adatom mobility is too low for surface diffusion to fill the voids [12]. Nanosphere templates are favorable to further enhance the atomic shadowing as nucleation sites for nanostructures. The substrate is shadowed during deposition, first by the array of nanospheres and then by the growing nanostructures. By causing the vapor flux to arrive at an extreme glancing angle (which is 85° in our work) and applying periodic polystyrene sphere arrays as templates, atomic shadowing is greatly enhanced and regular close-packed arrays of nanostructures can be engineered.

It is observed that the thickness of the nanoflakes in the shape of melon seeds, shown in Figure 3a, decreases as a function of height, which is attributed to the limited adatom surface diffusion and atomic shadowing effects. Besides, the length-width ratio of nanoflakes increases gradually with the enhancement of the discharge current. The average length-width ratios of the sample 1#-3# were measured through a number of micrographs and are outlined in Table 1.

**Table 1:** The average length-width ratios of the samples and corresponding parameters

Sample ID	1#	2#	3#
Discharge current (A)	0.15	0.20	0.25
Length-width ratio	1.19	1.53	2.29

The effect of the deposition parameter to the morphology of nanostructures was studied. As GLAD is a physical vapor deposition process in which the incident flux impinges the substrate from an oblique angle, causing atomic shadowing and resulting in highly porous nanostructures [13], the morphology of nanostructures is closely related to the direction and velocity of the incident flux. With the incident flux from lateral SnO_2 _target, the direction of the growth front was changed from perpendicular to lateral, leading to an increase of the growth rate in the direction parallel to the SnO_2 _flux and a decrease in the perpendicular direction [14]. As a result, the width of the nanostructures became broadened parallel to the SnO_2 _flux, and suppressed perpendicular to it, as shown in Figure 3a. The arrangement of the nanoflakes corresponds with the direction of SnO_2 _flux illustrated in Figure 1a, confirming that the nanostructures were deformed by the incident flux that caused anisotropic lateral growth. With the increased sputtering rate of the Ti target, the effect of lateral SnO_2 _flux was gradually overweighed by Ti flux, resulting in the increase of the length-width ratio. Thus, it can be concluded that the sputtering rates of Ti target and SnO_2 _target have a great influence on the morphology of nanostructures.

**Figure 4:**
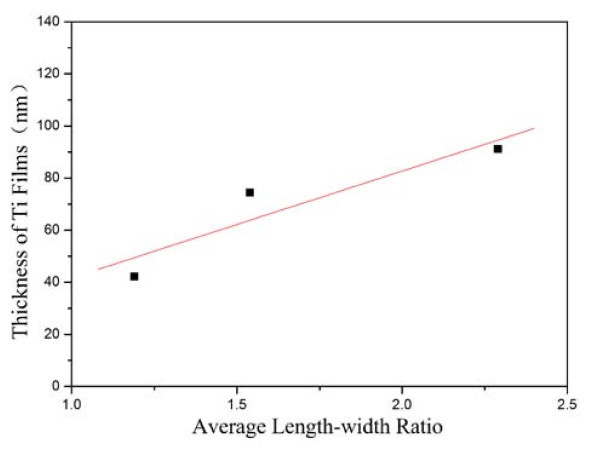
**The length-width ratio of nanostructures versus the thickness of corresponding Ti films**.

To investigate the relationship of the sputtering rate with the shape of nanostructures, the depositions of Ti films were carried out, with the discharge currents set to 0.15, 0.20, and 0.25 A, respectively, and other conditions consistent with the depositions of Ti_*x*_Sn_1-__*x*_O_2_. The thicknesses of the prepared Ti films for the three specimens are 42.3, 74.4, and 91.2 nm, respectively, measured through the SEM micrographs (the resolution of the applied scanning electron microscope is 2.0 nm). Because the thickness of the Ti films is directly proportional to the sputtering rate of the Ti target with other factors being held constant, the question can be transformed to the relationship of the thickness of Ti films with the length-width ratio of nanoflakes, as illustrated in Figure 4. This result confirms that the average length-width ratio of the nanostructures holds a linear relationship with the thickness of the Ti films, suggesting the linear dependence of the length-width ratio on the sputtering rate of Ti target. Accordingly, the morphology of the nanostructures can be modulated by regulating the sputtering power of Ti target.

**Figure 5:**
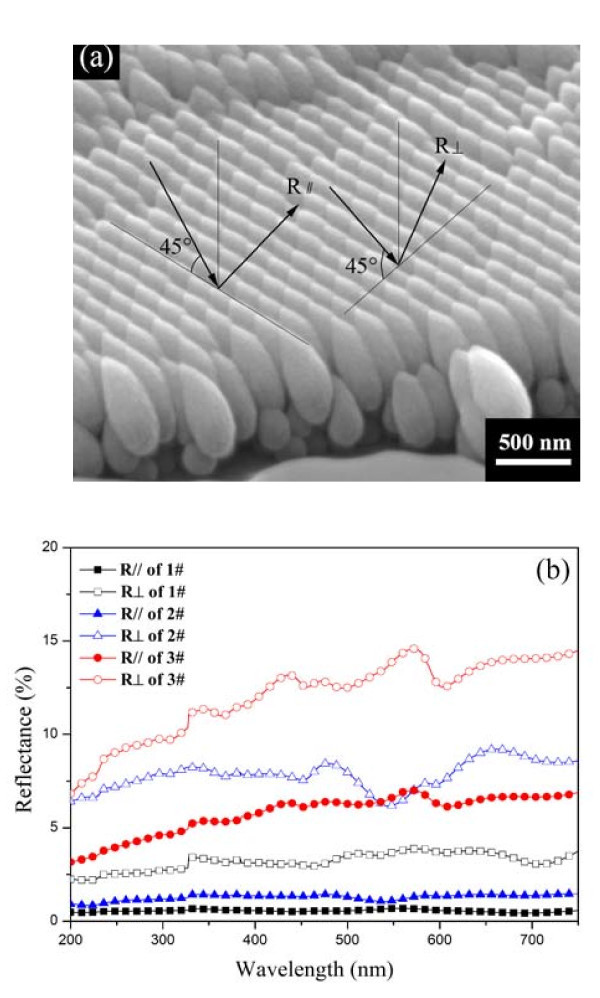
**(a) Schematic illustration of the incident and reflected lights, with the reflectance in two directions marked as *R*_*|| *_and *R*_⊥_, respectively; and (b) the *R*_*|| *_and *R*_⊥ _of samples 1#-3# at the wavelength in the range of 200-750 nm**.

In view of the anisotropic morphology of the nanoflakes, the anisotropism of the optical property was studied. The reflectances of samples 1#, 2#, and 3# were measured using a spectrophotometer. Figure 5a shows the directions of the incident light. The reflectance in the direction parallel to the surface of nanoflakes is marked as *R*_||_, while the reflectance in the other direction is marked as *R*_⊥_. Figure 5b provides the *R*_|| _and *R*_⊥ _of the samples 1#, 2#, and 3# in the spectral range of 200-750 nm. It indicates that the reflectance rises as the Ti content increases, and that the reflectance of sample 1# is almost wavelength independent, which agrees with the previously reported study on the optical properties of metal-dielectric composite media close to the percolation threshold [15-17]. In addition, it is notable that the reflectances of samples 1# and 2# are rather low, especially in the direction parallel to the surface of nanoflakes. This can be explained by the model of two-dimensional subwavelength antireflection nanogratings. The earlier report showed that the gradient-index layer may significantly influence the reducing reflection [18]. Hence, the gradient-duty cycle subwavelength nanogratings were designed to suppress the reflection, which functioned by providing a graded transition of the refractive index between air and the substrate [19-21]. Because of the gradient width and the thickening of the nanoflake with height, it can be approximately equivalent to a gradient-duty cycle subwavelength nanograting, which accounts for the effect of antireflection. Comparing the reflectance in two directions, it is evident that *R*_|| _is apparently lower than *R*_⊥ _for all the samples. This feature can be attributed to the anisotropism of the morphology, which plays a crucial role in the anisotropism of the optical property. It demonstrates that the preparation method we proposed in this article can accomplish an adjustment to the morphology of nanostructures, and ultimately the tuning of the properties.

## Conclusions

We have reported a general and an effective process for producing semiconductor oxide materials with controllable morphology and properties by glancing angle sputter deposition. Periodic arrays of Ti_*x*_Sn_1-__*x*_O_2 _nanostructures were prepared on patterned Si substrates through the co-sputtering of Ti and SnO_2_. The shape of Ti_*x*_Sn_1-__*x*_O_2 _nanostructures, characterized by the length-width ratio which linearly depended on the sputtering rate of Ti target, could be controlled by adjusting the sputtering power of Ti target. Optical properties were studied, and the results confirmed that the melon seed-shaped nanoflakes, which were approximately equivalent to gradient-duty cycle subwavelength nanogratings, possessed very low reflectance. Furthermore, their reflectances were anisotropic, which can be ascribed to the anisotropism of the morphology. Consequently, this study provides the opportunity to design optical devices by creating nanostructures with tailored morphology and unique performance.

## Abbreviations

GLAD: glancing angle deposition; SEM: scanning electron microscopy; XRD: X-ray diffraction.

## Competing interests

The authors declare that they have no competing interests.

## Authors' contributions

SC carried out the studies and drafted the manuscript. ZL participated in the design of the study and helped in revising the manuscript. ZZ participated in the design of the study and gave suggestions on the analysis of results. All the authors read and approved the final manuscript.
